# Maternal Smoking History Enhances the Expression of Placental Growth Factor in Invasive Trophoblasts at Early Gestation Despite Cessation of Smoking

**DOI:** 10.1371/journal.pone.0134181

**Published:** 2015-07-27

**Authors:** Akihiro Kawashima, Keiko Koide, Junichi Hasegawa, Tatsuya Arakaki, Shin Takenaka, Daisuke Maruyama, Ryu Matsuoka, Akihiko Sekizawa

**Affiliations:** Department of Obstetrics and Gynecology, Showa University School of Medicine, Shinagawa, Japan; University of Barcelona, SPAIN

## Abstract

Maternal smoking during early pregnancy is associated with a reduced risk for preeclampsia even after smoking cessation during pregnancy. Although the pathophysiology of preeclampsia has not been established, placental growth factor (PlGF) is believed to be a key factor. The aim of this study was to assess the effect of maternal smoking on the PlGF expression in invasive trophoblasts at early gestation. We collected villous tissues from women requesting surgical termination due to non-medical reasons at 7-8 weeks of gestation. The maternal smoking status was evaluated by measuring the serum cotinine level and patients were divided into two groups: active smokers and non-smokers. After separating invasive trophoblasts from villous tissues cultured initially under 2% O_2_ for 24 hours, the separated invasive trophoblasts were cultured under 2% or 8% O_2_ for 48 hours. The expression levels of the *PlGF* gene in villous tissue specimens and in invasive trophoblasts cultured after the conditions were quantified using qRT-PCR. The levels of PlGF protein in the medium were quantified using an ELISA. The gene expression level of *PlGF* in the villi in the active-smokers was significantly higher than that of the non-smokers. In comparison of the invasive trophoblasts under normoxia and oxygenated conditions, the ratio of *PlGF* gene expression and protein expression under oxygenation (2% O_2_+8% O_2_ / 2% O_2_+2% O_2_) in the active-smokers were both significantly higher than in the non-smokers. Maternal smoking history appears to stimulate PlGF expression in invasive trophoblasts under oxygenated conditions. This may be one of several causes leading to the protective effect of maternal smoking on preeclampsia.

## Introduction

Preeclampsia is a pregnancy-specific syndrome affecting 5% of all pregnancies and it is also a major cause of maternal mortality. The pathophysiology of preeclampsia remains largely unclear. Several pathophysiological studies have shown that preeclampsia is characterized by poor placentation with a shallow invasion of extravillous trophoblasts and an impaired spiral artery remodeling[1]. This invasion and remodeling begins late in the course of the first trimester[2]. Seminal work by Romeo et al.[3] showed that in preeclamptic women, the circulating levels of placental growth factor (PlGF), a pro-angiogenic placental factor, was already decreased from the first trimester. An imbalance between pro- and anti-angiogenic placental factors may contribute to the manifestations of preeclampsia.

It is notable that, in pregnancy, the levels of PlGF were found to be higher in smokers compared with non-smokers. It has been suggested that smoking may exert a protective effect by affecting this imbalance. It has also been acknowledged that exposure to tobacco during pregnancy modifies important aspects of the placental function. Previous studies have shown that there are placental complications linked to cigarette smoke exposure during pregnancy[4], including placental abruption[5], preterm delivery[6] and a reduction in the birth weight[7]. One prospective evaluation demonstrated that first trimester fetal growth was associated with smoking habits, and first trimester fetal growth restriction was associated with low birth weight and preterm birth[8]. Paradoxically, it has been noted that the risk of preeclampsia is lower in smokers than in non-smokers. This effect was verified by a number of groups[9, 10]. Additionally, some studies have consistently shown that women who quit smoking in early pregnancy had a reduced risk of preeclampsia[11, 12].

It is generally assumed that during the first half of pregnancy, the placenta’s own genetic program is the primary determinant of growth, while during the second half of pregnancy, many external factors, such as maternal factors, pregnancy-related factors and environmental factors have an increasing impact on the placental development[13–15]. The results of our previous study revealed that maternal smoking during the early first trimester affects the pro-angiogenic gene expression profiles of the villi in the first trimester[16]. In a recent study, maternal smoking appears to be involved in DNA methylation, which may subsequently affect the placental and fetal growth[17]. However, this epigenetic effect is not yet clear.

These data led us to hypothesize that the effect of maternal smoking on invasive trophoblasts will increase the *PlGF* gene expression and the PlGF protein excretion during the late first trimester, when the oxygen concentration in the intervillous spaces increases from 2% before 9 weeks of gestation to 8% at 10–12 weeks of gestation[18]. In this study, we focused on invasive trophoblasts from the first trimester villi and the oxygen level of the culture condition. The aim of this study was to increase our insight into the impact of maternal smoking on the subsequent placental function patterns with a particular focus on PlGF.

## Results

**Fig 1:**
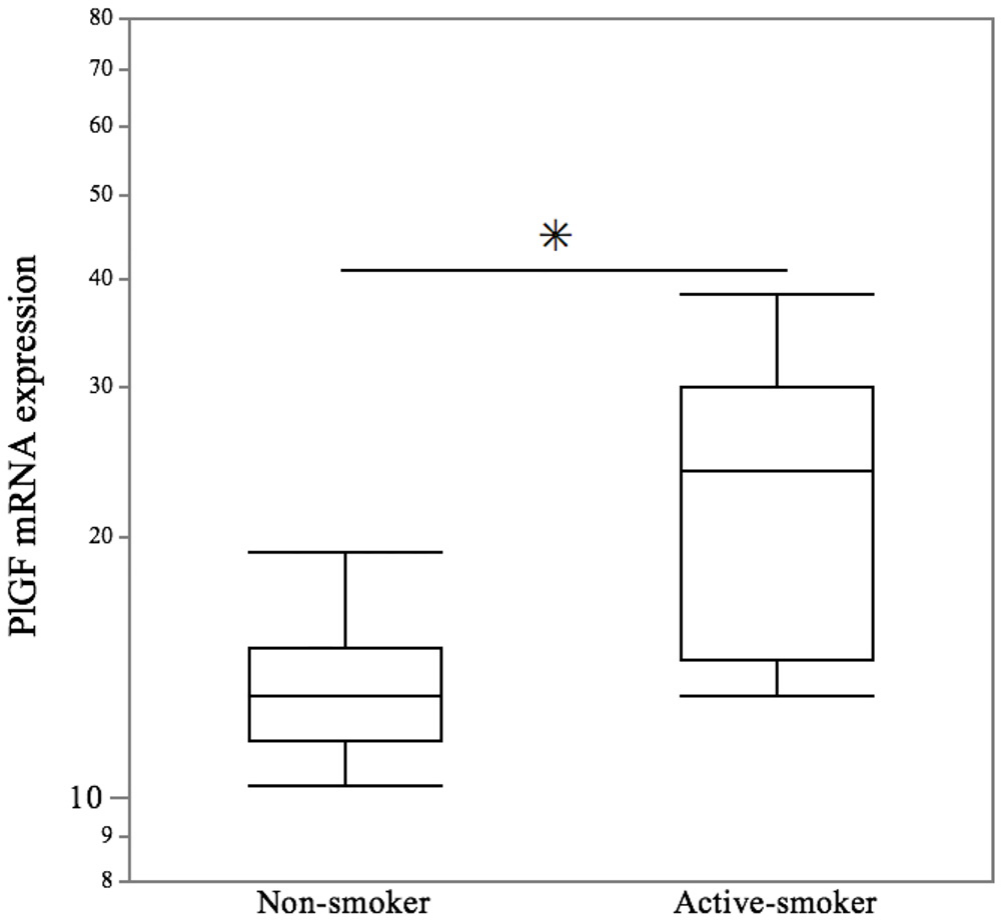
Effect of maternal smoking on the expression of the *PlGF* gene in villous tissues. Using real-time quantitative PCR analyses, the villous tissues in the active smoker group showed a significant increase in the gene expression of *PlGF* compared with the non-smoker group. The values are relative to the expression of *ACTB*. The central bars represent the median values, boxes represent the interquartile ranges and whiskers represent the 90th and 10th percentiles. * *p*<0.05, in the comparison between the non-smokers and active-smokers.

The clinical background available for each study group, including maternal age, maternal body weight, BMI, the self-reported smoking status and the cotinine concentrations are presented in Table 1. There were no significant differences in maternal age, BMI and the crown-rump length between the two groups. However, there were significant differences in the self-reported smoking status and serum cotinine levels between the two groups. To determine the effect of villi exposed to maternal smoking on the *PlGF* gene expression, the levels of *PlGF* gene expression in the villi from active smoking mothers were compared to non-smoking mothers. The *PlGF* transcripts in villi were significantly higher in the active smoking group compared with the non-smoking group (*p* = 0.011) (Fig 1).

**Table 1:** The background and cotinine levels in the study group.

	Non-smokers (n = 11)	Active-smokers (n = 8)	*p* value
Age (years)	37 (23–41)	32 (26–34)	0.199 ^a^
Body weight (kg)	50 (48–58)	52 (45.5–54.5)	0.901 ^a^
BMI	20.3 (20.0–22.2)	19.8 (19.2–20.4)	0.216 ^a^
Self-reported smoker (n)	0 (0%)	5 (62.5%)	0.005^b^
Serum cotinine (ng/mL)	0.3 (0.2–0.4)	130.8 (36.9–145.8)	<0.001 ^a^
Crown-rump length	14 (11–17)	15 (10–16)	0.772 ^a^

There were no significant differences in the maternal age, BMI, or crown-rump length between the non-smokers and the active smokers. The data are presented as the median and quartiles.

^a^ *p* values obtained using the Wilcoxon-Mann-Whitney method.

^b^ *p* values obtained using Fisher’s exact test.

**Fig 2:**
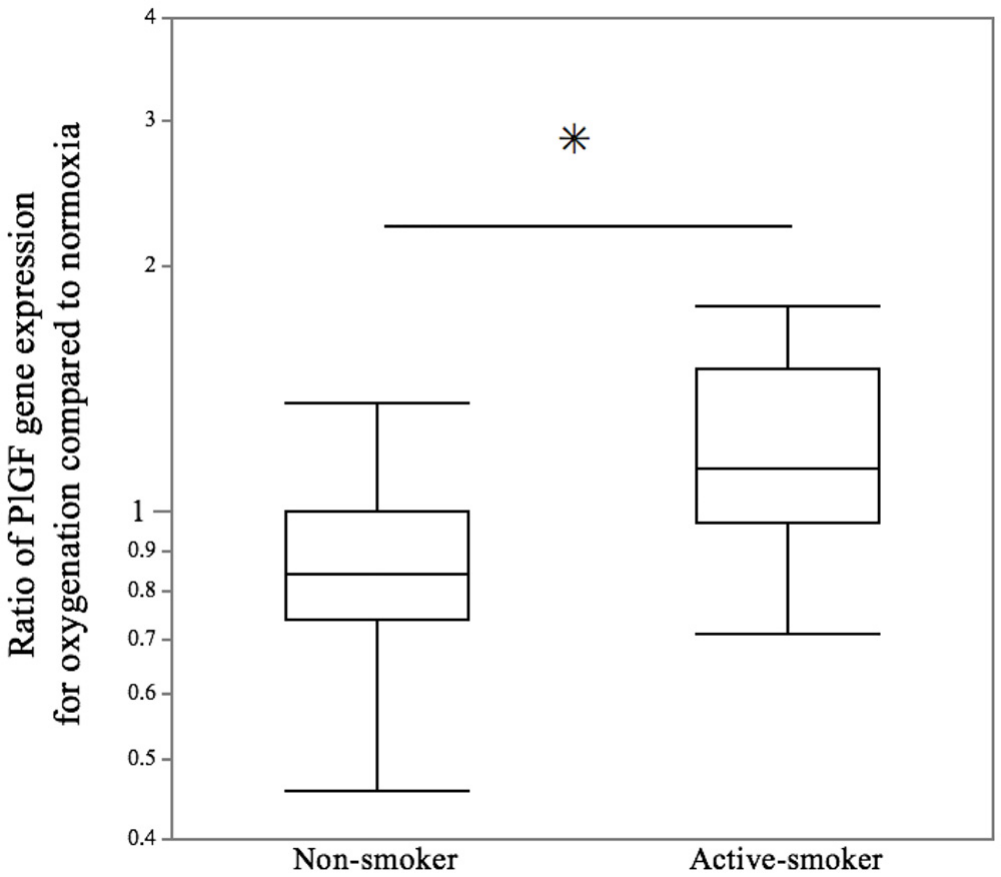
Ratio of *PlGF* gene expression in invasive trophoblasts under oxygenation compared to normoxia conditions. Using real-time quantitative PCR analyses, the ratio of *PlGF* gene expression in invasive trophoblasts under oxygenation in the active-smoker group was compared with the non-smoker group. The central bars represent the median values, boxes represent the interquartile ranges and whiskers represent the 90th and 10th percentiles. *p<0.05, in the comparison between the non-smokers and active-smokers.

**Fig 3:**
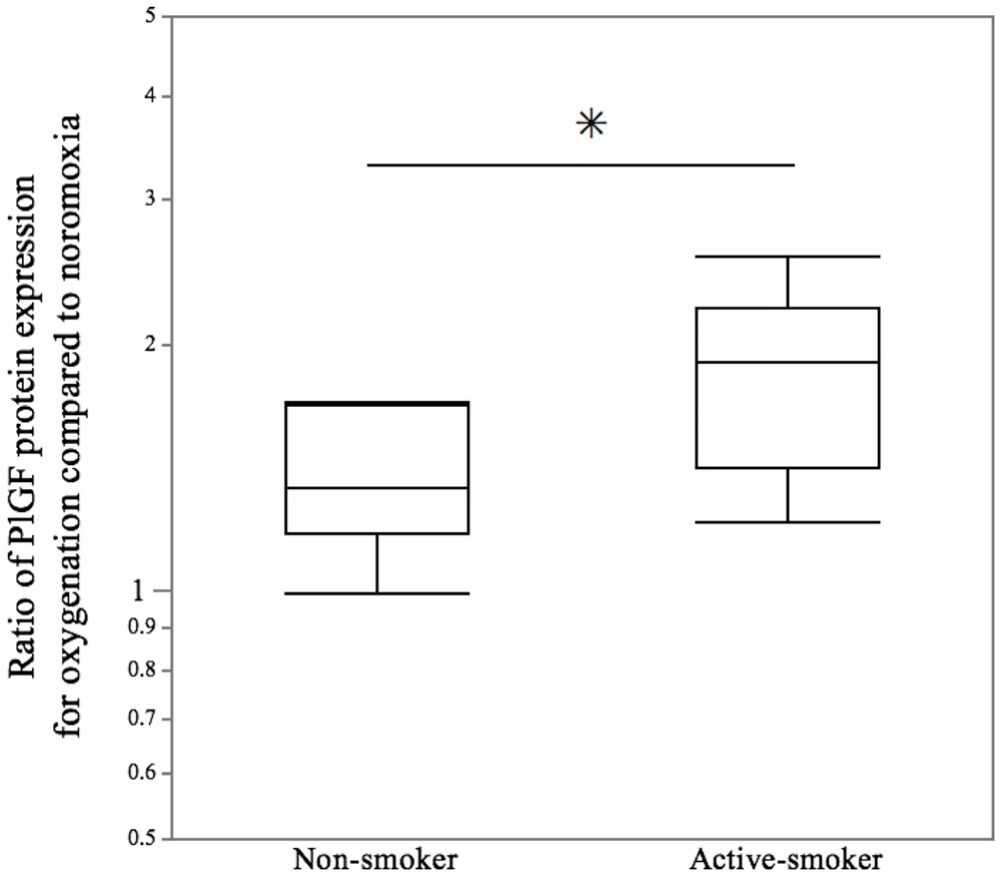
Maternal smoking enhanced the ratio of PlGF protein in invasive trophoblasts under oxygenation compared to normoxia conditions. Using an ELISA analysis, the ratio of PlGF protein under oxygenation in the active-smoker group was found to be significantly increased compared with the non-smoker group. The central bars represent the median values, boxes represent the interquartile ranges and whiskers represent the 90th and 10th percentiles. * *p*<0.05, in the comparison between the non-smokers and active-smokers.

**Fig 4:**
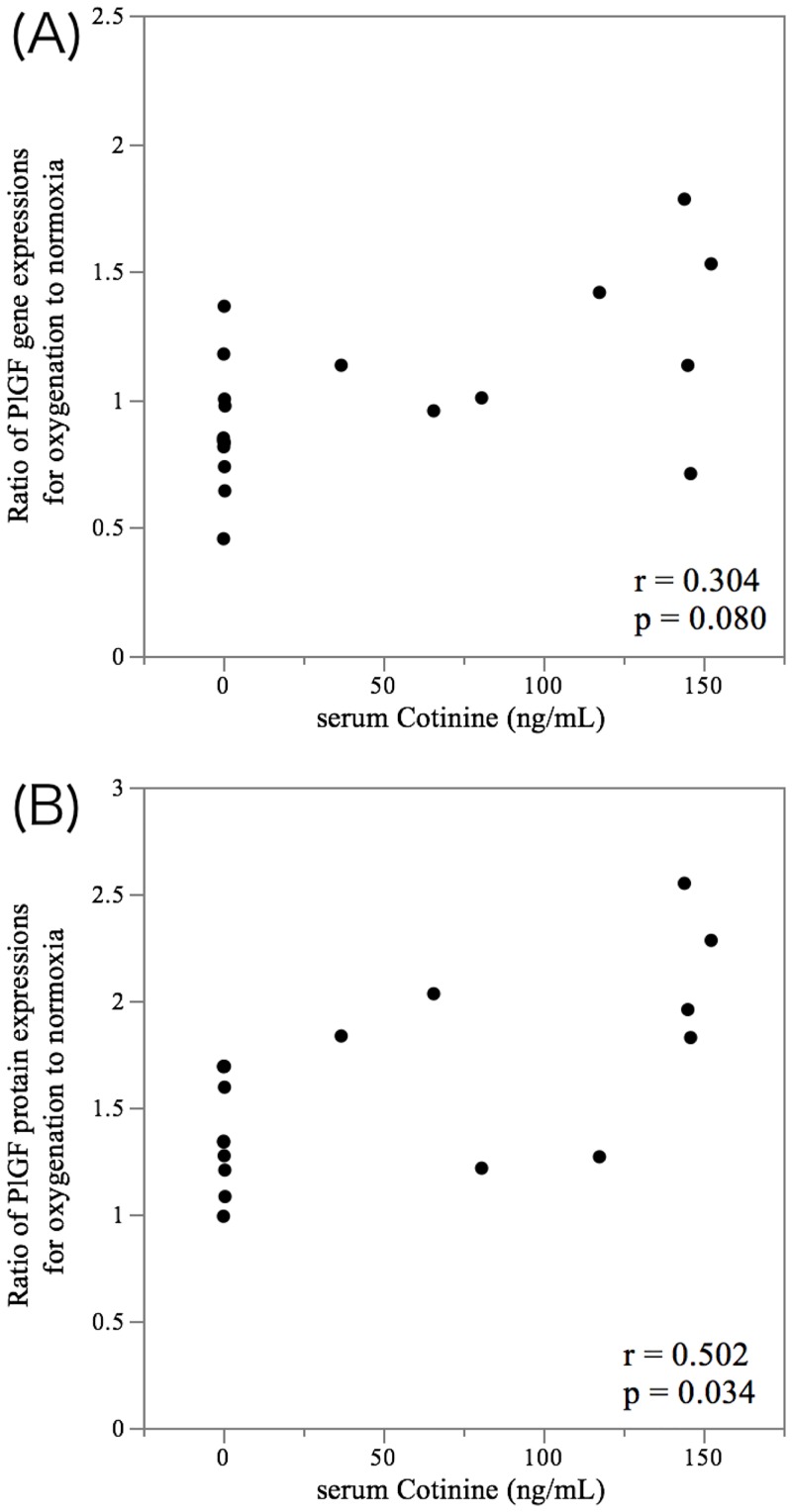
Correlation between the serum cotinine levels and the ratio of *PlGF* gene expression and protein under oxygenation. Scatter plots show the correlations between the serum cotinine levels and the ratio of *PlGF* gene expressions (A) and the ratio of PlGF protein expressions (B) in invasive trophoblasts during oxygenation compared with normoxia conditions. The significance was calculated according to Spearman’s correlation.

To further investigate whether exposure from maternal smoking could upregulate PlGF expression after cessation, the influence of the history of maternal smoking during increasing oxygenation on the levels of *PlGF* transcript and PlGF protein in invasive trophoblasts was examined under normoxia and oxygenated conditions. The ratio of *PlGF* gene expression for oxygenation (2% O_2_+8% O_2_ / 2% O_2_+2% O_2_) in invasive trophoblasts exposed to maternal smoking showed a median fold change of 1.13 (interquartile range [IQR], 0.97 to 1.50), while in invasive trophoblasts unexposed to maternal smoking, the ratio of the gene expression showed a median fold change of 0.84 (IQR, 0.74 to 1.00) (*p* = 0.039) (Fig 2). Furthermore, there was a dramatic increase in the ratio of PlGF protein excretion during oxygenation. The ratio of PlGF protein excretion during oxygenation in invasive trophoblasts exposed to maternal smoking showed a median fold change of 1.90 (IQR, 1.41 to 2.22), while the ratio in invasive trophoblasts unexposed to maternal smoking showed a median fold change of 1.34 (IQR, 1.18 to 1.69) (*p* = 0.021) (Fig 3). While there was no correlation between the serum cotinine levels and the ratio of *PlGF* gene expression during oxygenation (*r* = 0.304, *p* = 0.080) (Fig 4A), a significant relationship between the serum cotinine levels and ratio of PlGF protein expression during oxygenation was observed (*r* = 0.502, *p* = 0.034) (Fig 4B).

## Discussion

Our results indicate that exposure to maternal smoking significantly increased the gene expression of *PlGF* in the early first trimester. Furthermore, the exposure to maternal smoking increased the gene expression and the secretion of PlGF during oxygenation in invasive trophoblasts from early first trimester villi. These findings suggest that exposure to maternal smoking in the first trimester may increase the PlGF serum levels, even after smoking cessation, and prevent the incidence of preeclampsia.

PlGF is a major member of the vascular endothelial growth factor family, which is a ligand for vascular endothelial growth factor receptor (VEGFR1) that enhances the angiogenic response of vascular endothelial growth factor (VEGF. PlGF is related to VEGF-A and binds to VEGFR1). PlGF enhances VEGF-induced angiogenesis and permeability[19, 20]. The physiological role of PlGF is less well known than that of VEGF, however, PlGF appears to stimulate angiogenesis under conditions of ischemia, inflammation, and wound healing[21]. PlGF reportedly causes microvascular relaxation of rat renal arterioles *in vitro* which is blocked by sFlt-1[22].

Decreased circulating free PlGF is observed in women diagnosed with preeclampsia and circulating levels of PlGF are altered before the onset of clinical symptoms[23]. Whether this decrease is secondary to reduced PlGF biosynthesis or reflects excess anti-angiogenic molecules, such as soluble fms-like tyrosine kinase-1 (sFlt-1), in the circulation of preeclamptic patients is still not clearly understood[24]. The PlGF levels are lower in preeclamptic patients before the sFlt-1 levels change[25]. In normal pregnancies, circulating PlGF increases from the first trimester up to 32 weeks of gestation[26, 27]. A recent study indicated that oxygen is the main regulator of the expression of PlGF. The expression of PlGF in trophoblasts is stimulated by high oxygen concentrations and reduced by low oxygen concentrations[28], which is supported by the findings of the present study.

The biological mechanism underlying the prevention of preeclampsia by cigarette smoke has not yet been established. Cigarette smoke has been shown to increase the *PlGF* gene expression in a trophoblast cell line. A recent study suggested that sFlt-1 suppresses endothelial fundamental functions for angiogenesis, and that nicotine, a major component of cigarette smoke, restores these functions possibly through increasing the secretion of PlGF[29]. The present study supported our previous study that maternal smoking increased the *PlGF* gene expression of villi in the first trimester in another cohort[16]. These findings are consistent with other groups who have reported that maternal smoking was associated with modified gene expression[16, 30, 31]. The placenta plays an important role in its own development as it produces a number of growth factors. The placenta exhibits a significant degree of metabolic activity, including the mechanism of toxic compounds[32]. Many toxicants act directly or potentially by altering the metabolic functions of the placenta. Environmental toxicants may affect the placental function by modifying the epigenetic state of the tissue, including altering DNA methylation[33]. Maternal tobacco smoking during pregnancy is associated with altered epigenetic mechanisms, such as DNA methylation patterns in the placenta[34, 35]. The life-long effects of prenatal exposure to tobacco smoke increase the risk for developing diseases later in a child’s life, which may be mediated through changes in DNA methylation[36]. In the present study, the gene expression and the protein secretion of PlGF in invasive trophoblasts exposed to maternal smoking under oxygenation were higher than in invasive trophoblasts unexposed to maternal smoking, suggesting that maternal smoking during the early first trimester increases PlGF even after smoking cessation. These results indicate that maternal smoking during the first trimester may alter the epigenetic mechanisms.

Our study focused on the gene expression and the protein excretion of PlGF and did not resolve the epigenetic effect associated with increasing *PlGF* gene expression. Future studies are warranted to determine the precise relationship. Another limitation is our small sample sizes. Small sample size (n = 8 for the active smokers group) may explain why no correlation the serum cotinine levels and the ratio of *PlGF* gene expression during oxygenation. A larger sample size would help to investigate the long term alterations in PlGF of previous exposure to smoking at the first trimester.

The results of the present study indicate that differences in the rates of change of *PlGF* gene expression and protein excretion between the study groups are already detectable in the first trimester of pregnancy in spite of our small samples, which is in line with our hypothesis that these differences are the result of epigenetic modifications in the placenta after maternal smoking exposure. Further investigations on the mechanism of this effect in the epigenetic changes, DNA methylation, chromatin remodeling and microRNA for the regulating of angiogenic factors are currently being pursued.

## Materials and Methods

### Tissue collection

Invasive trophoblasts obtained from surgically removed villous tissue specimens obtained from pregnant females requesting artificial abortion before 7 weeks of gestation were analyzed between February 2014 and July 2014. The villous tissue specimens obtained from cases of multiple gestation, illicit drug use and preexisting medical conditions, such as diabetes, chronic hypertension and renal disease, were excluded in the present analysis. The gestational age was confirmed by ultrasound measurements of the crown-rump length with detection of the fetal heartbeat at Okayama Clinic (Tokyo, Japan) and Kitamura Clinic (Kawasaki, Japan), and villous samples were collected at 7–8 weeks (n = 21). This study was approved by the Ethics Committee of Human Genomic Analysis at Showa University School of Medicine (144/2011). Written informed consent was obtained from each patient prior to participation. Following correction, the villi were immediately suspended in sterile saline and transported to the laboratory at the Department of Obstetrics and Gynecology at Showa University School of Medicine (Tokyo, Japan) within three hours, at which time the villous tissues were washed three times in sterile PBS to remove excess blood.

### Blood sampling and cotinine analysis

Prior to pregnancy termination, blood samples were collected from the patients and centrifuged at 1,600xg for 10 min at 4°C. The resulting serum was transferred into plain polypropylene microtiter plates. The serum cotinine levels were quantified by using an enzyme-linked immune absorbent assay (ELISA; Cosmic Corporation, Tokyo, Japan) that had a detection limit of 0.6 ng/mL and an inter-assay variation of < 7%. The participants were categorized as non-smokers if their serum cotinine levels were < 1.0 ng/mL (n = 11) or as active smokers if their levels were > 5.3 ng/mL (n = 8), since a serum cotinine cut-off value of 3.0–5.3 ng/mL was previously recommended to separate smokers from non-smokers[37, 38]. Patients whose serum cotinine levels ranged between 1.0–5.3 ng/mL were excluded from this study (n = 3).

### Placental sample collection

The villous tissue was separated from the decidua using light microscopy. A portion of the tissue sample was transferred to a tube containing 1.0 mL of RNAlater solution (RNA stabilization reagent; Qiagen, Hilden, Germany) and was stored overnight at 4°C. After the reagent was removed, the samples were stored at -80°C until RNA isolation.

### Isolation, purification and culture of invasive trophoblasts

Invasive trophoblasts were isolated from the villous tissues using a previously published method[39–41]. Briefly, the residual villous materials after placental sample collection were washed in Hank’s balanced salt solution (HBSS, Sigma-Aldrich Co., St. Louis, MO) and minced to a size of 0.5 mm^3^ and digested for 35 minutes at 37°C in 0.25% trypsin (Invitrogen Co., Carlsbad, CA) and 0.5 mg of DNase I (Sigma-Aldrich Co., St. Louis, MO) twice. At the end of the warm extraction procedure, the supernatants were collected. Following centrifugation, the cell pallets were resuspended in HBSS, loaded on top of a 5% step-layer Percoll (Sigma-Aldrich Co., St. Louis, MO) gradient ranging from 10–70%, and centrifuged. The trophoblasts were isolated from the middle layer of 35–45% Percoll and cultured within culture medium (Ham’s F12 supplemented with 10% FBS, 1,000 U/ml of penicillin, 1 mg/ml of streptomycin and 1.5 mg/ml of amphotericin B obtained from Sigma-Aldrich Co., St. Louis, MO). The trophoblasts (5 x 10^5^ cells/500 μL per well) were plated in a 24-well plate coated with growth factor reduced Matrigel (Becton Dickson, East Rutherford, NJ) and incubated for 24 hours at 37°C, 5% CO_2_ and 2% O_2_ to promote the invasion of invasive trophoblasts into the Matrigel. After 24 hours, the medium was changed, and the invasive trophoblasts were subsequently cultured under 2% O_2_ (normoxia condition; 2% O_2_ + 2% O_2_) and 8% O_2_ (oxygenated condition; 2% O_2_ + 8% O_2_) for 48 hours. At the end of the incubation periods, the spent media were collected, centrifuged to remove debris, aliquoted, and stored at -20°C until assayed, and the cells were removed from the Matrigel after spreading cell recovery solution (BD Biosciences, Bedford, MA). The layers of cells and gel were then scraped with 2 ml of cell recovery solution per well, and the samples were incubated on ice for one hour until the Matrigel was completely dissolved, after which the cells were recovered via centrifugation (300xg for five minutes at 4°C) and washed twice in sterile PBS.

### RNA extraction and reverse transcription

Total RNA was extracted from the cell pellets using the RNeasy Mini Kit (Qiagen, Valencia, CA) according to the manufacturer’s instructions. The purity of the RNA was determined using a NanoDrop ND-1000 spectrophotometer (Thermo Scientific Inc., Wilmington, DE) by measuring the absorbance at 260 and 280 nm. An OD260/280 ratio greater than 1.90 was considered to indicate that the sample was acceptable for further processing. All RNA samples met this purity requirement. The extracted total RNA (2 μg) was immediately reverse transcribed into cDNA using the PrimeScript RT Master Mix (Takara Bio Inc., Shiga, Japan), according to the manufacturer’s instructions. The process was performed in a Veriti Thermal Cycler (Applied Biosystems, Foster City, CA) under the following thermal conditions: 15 minutes at 37°C, followed by five minutes at 85°C.

### Quantitative RT-PCR

A real-time quantitative PCR analysis was performed using the StepOnePlus Real-Time PCR System (Applied Biosystems, Foster City, CA). The Assay-on-Demand TaqMan primer, PlGF (TaqMan Gene Expression Assay ID Hs00182176_m1) from Applied Biosystems was used to amplify PlGF. *ACTB* (Hs01060665_g1) was used as the reference gene. The thermal cycling conditions were as follows: 95°C for 30 sec, followed by 40 cycles of 95°C for five sec and 60°C for 30 sec. All samples were analyzed in duplicate, and multiple negative water blanks were included in each analysis. The transcript numbers were determined from the linear regression of the standard curves. The *PlGF* gene expression levels were normalized to the levels of *ACTB*, and the relative gene expression was reported as a ratio.

### PlGF ELISA Assay

The concentrations of PlGF were measured from culture medium using a specific PlGF ELISA kit (R&D Systems, Minneapolis, MN) following the manufacturer’s instructions. This kit recognizes recombinant and natural human PlGF, and no significant cross-reactivity or interference was observed with VEGF or PDGF. Briefly, 100 μL conditioned medium and 100 μL Assay Diluent were added to each well and the samples were incubated for two hours at room temperature. After the samples were washed four times, 200 μL PlGF Conjugate buffer was added and the samples were incubated for one hour. After repetitive washing, 200 μL Substrate Solution was added for 30 min. Finally, 50 μL Stop Solution was added and the optical density was determined using a microplate reader set at 450 nm. A standard curve was obtained by serial dilution of PlGF. This experiment was repeated in triplicate in three cultures.

### Data analysis

The ratios of *PlGF* gene expression and PlGF protein expression under oxygenation compared with normoxia conditions were computed by dividing the individual *PlGF* gene expression and PlGF protein expression under oxygenation by the individual *PlGF* gene expression and PlGF protein expression in normoxia conditions. The data are presented as medians (interquartile ranges [IQRs]). Spearman’s correlation coefficients were used to determine the relationship between the serum cotinine levels and the ratio of *PlGF* gene expression and PlGF protein expression under oxygenation compared with normoxia conditions. Statistically significant differences were assessed using the Wilcoxon-Mann-Whitney test. Categorical variables were compared by Fisher’s exact test. All analyses were conducted using the JMP version 11.0.2 software program (SAS Institute, Cary, NC). A p value of <0.05 was considered to be statistically significant.
