# Expression of Concern: Maternal Smoking History Enhances the Expression of Placental Growth Factor in Invasive Trophoblasts at Early Gestation Despite Cessation of Smoking

**DOI:** 10.1371/journal.pone.0279474

**Published:** 2023-01-11

**Authors:** The PLOS ONE Editors

The Funding Statement for this article [1] states that the study received funding from the Smoking Research Foundation, which according to [2] has received financial support from the tobacco industry. In light of this issue the *PLOS ONE* article [1] does not comply with the journal’s policy on Funding from Tobacco Companies [3] which was implemented in 2010.

Therefore, the *PLOS ONE* Editors issue this Expression of Concern.

We regret that this concern was not identified and addressed prior to the article’s [1] publication.

## References

[1] Kawashima A, Koide K, Hasegawa J, Arakaki T, Takenaka S, Maruyama D, et al. (2015) Maternal Smoking History Enhances the Expression of Placental Growth Factor in Invasive Trophoblasts at Early Gestation Despite Cessation of Smoking. PLoS ONE 10(7): e0134181. doi: 10.1371/journal.pone.0134181 26214510 PMC4516258

[2] Iida K, Proctor RN. (2018) ‘The industry must be inconspicuous’: Japan Tobacco’s corruption of science and health policy via the Smoking Research Foundation. Tobacco Control 27:e3–e11. doi: 10.1136/tobaccocontrol-2017-053971 29437992 PMC6073917

[3] https://journals.plos.org/plosone/s/disclosure-of-funding-sources#loc-funding-from-tobacco-companies

